# Exploring barriers to and motivations for vaccine uptake in a typhoid vaccine trial in Vellore, South India: a qualitative study

**DOI:** 10.1186/s12889-026-26362-z

**Published:** 2026-01-31

**Authors:** Nimi Elizabeth Thomas, B. L. Thabitha Malini, P. Dileepan, Jacob John

**Affiliations:** 1 Department of Community Medicine, Christian Medical College, Vellore, Tamil Nadu 632002 India; 2 Department of Rural Unit for Health and Social Affairs (RUHSA), Christian Medical College, Vellore, India 632209 Tamil Nadu

**Keywords:** Barriers, Facilitators, Vaccine hesitancy, Post pandemic, COVID-19, Typhoid conjugate vaccine, Clinical trial, Vaccine acceptance

## Abstract

**Background:**

Typhoid poses a significant challenge in India, which has one of the highest burdens of the disease globally, making the implementation of cost-effective vaccination strategies essential. The success of typhoid vaccination, whether through clinical trials or large-scale public health programmes, depends on community acceptance. In the post-COVID-19 period, vaccine hesitancy and scepticism have emerged as key challenges to both vaccine trials and mass vaccination efforts. This study examines the barriers to and motivations for vaccine uptake during a mass vaccination campaign.

**Methods:**

We conducted 15 focus group discussions (FGDs) and five in-depth interviews (IDIs) with participants and guardians from a cluster-randomised typhoid vaccine trial in Vellore, India. Data collection occurred two months after the vaccination campaign in purposively selected low- and high-coverage areas. FGD categories were determined a priori based on age, gender, and vaccination status, while IDIs were conducted when FGDs were not feasible. Data were collected until thematic saturation was achieved. The interviews were audio-recorded, transcribed, and analysed using thematic analysis.

**Results:**

Vaccination decisions were shaped by a combination of safety concerns, risk perceptions, and social influences. Key barriers included fears of long- and short-term adverse events, low perceived disease risk and consequently a reduced perceived need for vaccination, shifting attitudes toward new and adult vaccination following COVID-19, sociocultural beliefs, objections from household decision-makers, perceived lack of visible government endorsement and participation, and trust in alternative systems of medicine. The motivators included increased disease salience after the COVID-19 pandemic, increased perceived disease risk, positive influence from family members and peers, and trust in healthcare providers and professional recommendations. Study-related benefits, such as access to vaccines, follow-up, and free vaccination, operated bidirectionally, motivating many participants while raising concerns about vaccine quality among a few.

**Conclusion:**

In the post–COVID-19 era, vaccine availability alone does not ensure uptake. Vaccination programmes should actively engage household decision-makers, leverage trusted healthcare providers, address safety concerns and misconceptions surrounding new and free vaccines, and strengthen community trust alongside logistical planning.

**Supplementary Information:**

The online version contains supplementary material available at 10.1186/s12889-026-26362-z.

## Background

Typhoid fever, a severe systemic illness caused by *Salmonella enterica* serovar Typhi, continues to pose a significant global health threat with an estimated 7.15 million new cases and 93,300 deaths in 2021 [1]. This burden falls disproportionately on South Asia, Southeast Asia and sub-Saharan Africa, which account for over 90% of global cases [1, 2]. The burden is further compounded by the emergence of extensively drug-resistant *Salmonella* typhi strains [3], which threatens to undermine existing treatment strategies and highlights the vulnerability of populations in endemic areas. This disparity highlights the urgent need for effective control measures, particularly in resource-limited settings. India, with its high population density and challenges in water, sanitation, and hygiene (WASH) infrastructure, bears a substantial portion of the global typhoid burden [4, 5]. Within India, the city of Vellore has reported an alarmingly high incidence rate of typhoid among children, reaching 1,173 cases per 100,000 child-years [4].

Addressing the typhoid challenge necessitates a multifaceted approach that combines improvements in WASH infrastructure, access to effective and affordable diagnostics, timely diagnosis, and effective treatment. Vaccination stands out as a crucial strategy in combating typhoid, offering a proactive and cost-effective solution for disease control [6]. The World Health Organisation (WHO) recommends including a single dose of typhoid conjugate vaccine (TCV) in routine immunisation programmes in countries with a high burden of typhoid [7]. Evidence from Pakistan and Zimbabwe demonstrates the high effectiveness of TCV in controlling typhoid outbreaks and preventing infections caused by extensively drug-resistant strains [8, 9]. These successes highlight the potential of TCV to significantly reduce the global typhoid burden and protect vulnerable populations.

Despite compelling evidence supporting vaccination as a cornerstone of typhoid control, vaccine uptake presents a complex and evolving challenge, particularly in the wake of the COVID-19 pandemic [10]. The pandemic, while highlighting the importance of vaccines, also fuelled a wave of misinformation and mistrust [11], contributing to vaccine hesitancy. This hesitancy, often rooted in concerns about vaccine safety, efficacy, and necessity, has been identified by the WHO as a major threat to global health [12], jeopardising decades of progress in disease prevention and control.

A study in India highlighted the desirability of the typhoid vaccine in a trial context [13], but research on its broader acceptability across diverse real-world settings is limited. Although some quantitative studies have investigated factors affecting typhoid vaccine acceptance [14, 15] in similar low and middle-income settings, there is a critical need for qualitative research to uncover the deeper personal beliefs, social dynamics, and contextual factors that shape vaccine decision-making among healthy individuals and caregivers across different age groups following the COVID-19 pandemic. While post–pandemic research has largely focused on COVID-19 and childhood vaccines, there is limited qualitative evidence on adult decision-making for non-COVID vaccines. Behavioural frameworks such as the Health Belief Model (HBM) [16] and the WHO Behavioural and Social Drivers (BeSD) of Vaccination framework [17] offer useful conceptual structures for organising key influences on vaccine acceptance; however, these models require grounding in empirical, real-world evidence to capture how such factors are experienced and negotiated in specific contexts.

In our setting, we observed limited uptake of typhoid conjugate vaccines, especially among adults, despite a strong history of community engagement. This highlights the urgent need to understand the underlying perceptions that influence vaccine uptake, especially in anticipation of the potential inclusion of typhoid conjugate vaccines in the national immunisation programme and broader public sector rollout. This study aims to identify the primary barriers to vaccine uptake in Vellore, the key motivations driving uptake, and how these factors differ across demographic groups, including age, gender and vaccination status.

## Methodology

### Study setting

This qualitative descriptive study was conducted alongside an observer-blinded, cluster-randomised trial in Vellore, Tamil Nadu, South India, aimed at evaluating the effectiveness of the licenced typhoid conjugate vaccine, TyphiBEV®. Detailed descriptions of the trial design and procedures have been published previously [18]. The trial was conducted within the Vellore Health and Demographic Surveillance System and included participants aged 1 to 30 years. The trial is conducted by Christian Medical College (CMC), a 3675-bed multi-speciality hospital with a strong community reputation, providing subsidised care through two satellite hospitals and a study clinic in the study area. Willingness to vaccinate was not a prerequisite during recruitment.

Participants from the vaccine clusters were invited to receive the vaccine from predetermined clinics. Eleven centres, including CMC and its subsidiary hospitals, government primary healthcare centres (PHCs), and a private rehabilitation centre, were operational during the campaign. To increase participation, the campaign involved four months of door-to-door and street-level efforts, awareness activities led by medical professionals, engagement with community and religious leaders, and logistical support, including transportation to vaccination sites. This process was repeated after a secondary recruitment drive to include newly eligible participants and ensure coverage of those previously eligible. Despite these efforts, not all eligible participants were willing to receive the vaccination. Two months after the secondary vaccination campaign, we conducted this qualitative study using focus group discussions (FGDs) and in-depth interviews (IDIs) to explore barriers and motivations for vaccine uptake among the population who were offered the vaccine.

The study was approved by the Institutional Review Board and Ethics Committee of CMC, Vellore (CMC/IRB/0624128). We have reported our methods and findings in accordance with the Consolidated Criteria for Reporting Qualitative Studies (COREQ) guidelines (see Additional File 1) [19].

### Sampling

**Fig. 1:**
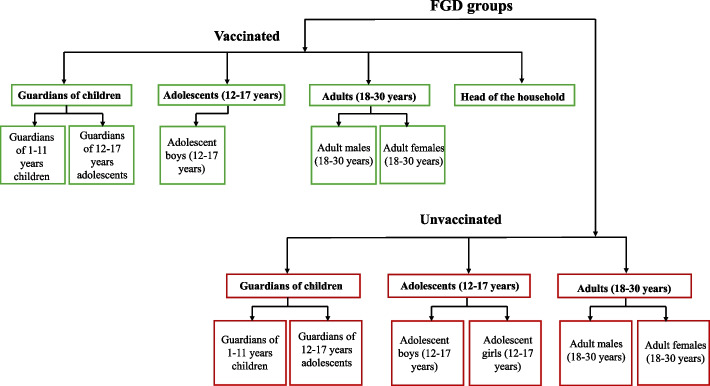
Overview of participant groups included in focus group discussions (FGDs) stratified by vaccination status, age, gender, and household role. Groups not represented in FGDs were included through in-depth interviews (IDIs); vaccinated adolescent girls were not included due to difficulties in mobilisation. Green represents vaccinated participants, and red represents unvaccinated participants

We used purposive sampling to achieve maximum variation while selecting the participants for the study. The participants included primary caregivers of children aged 1–11 years, primary caregivers of 12–17 years, adolescents aged 12–17 years (both males and females), adults aged 18–30 years (both males and females), and heads of household, with further stratification into TCV vaccinated and unvaccinated subgroups (Fig. 1). We purposively selected vaccinated participants from high-coverage areas (≥ 50% vaccination coverage in the relevant demographic group) and unvaccinated participants from low-coverage areas (< 50% coverage). Heads of households were chosen from households where no members or all members were vaccinated. Vaccinated participants were eager to join the FGDs, whereas unvaccinated individuals showed significant reluctance to participate. IDIs were conducted in areas where participants were reluctant to attend FGDs; these interviews included heads of household and adolescent girls, and all of them were unvaccinated. The thematic guide was developed, translated to the local vernacular (Tamil), and pilot-tested among vaccinated and unvaccinated participants to improve question framing and sequencing, ensuring that they were non-leading and non-judgmental to encourage honest responses (see Additional file 2). These pilot interviews are included in the results. The same guide was used for both FGDs and IDIs.

### Data collection

Data collection was conducted over a period of four months. The participants were identified through door-to-door visits in the study area, and those willing to participate were invited to a local venue at a mutually convenient time. All the interviews were conducted in Tamil language. The facilitator provided a verbal explanation of the study before the participants signed the consent forms, and demographic information was collected. Assent forms were obtained from children under 18 years of age, along with a parental signature for consent. To build rapport, warm-up questions were asked prior to the main interview questions. Interviews were audio-recorded with participant consent, and field notes were taken by a notetaker. FGDs were conducted separately for those who accepted and those who refused the vaccine, with additional consideration for gender and age to maintain group homogeneity. The discussions were held in a quiet community area, moderated by the second and third authors, both native Tamil speakers with a master's degree in social work, trained in qualitative data collection, and matched in gender with the participants. They had no prior contact with the participants. Personal identifying information was removed from the transcripts, and all audio recordings, field notes, and transcriptions were assigned unique identifiers. The FGD categories were determined a priori to capture shared norms and group dynamics across key demographic strata (age, gender, vaccination status, and household role), while IDIs were conducted for groups that were difficult to convene in group settings or where participants were reluctant to participate in FGDs. FGDs were repeated in selected participant groups when initial discussions did not yield sufficient information. Data collection was stopped when no additional themes emerged, and saturation was achieved.

### Data analysis

All the interviews were translated into English by a professional transcriber. The transcripts were checked against audio recordings by the investigators for quality control, and any discrepancies were resolved through consultations with the transcriber.

We used a constructivist approach and applied a six-step thematic analysis [20] to inductively code the transcripts which were manually uploaded in Microsoft Excel. Data were analysed using the Framework Method [21]. The first five transcripts were coded line by line using open coding, and the resulting codes were grouped into categories, leading to the development of an analytical framework. This framework was refined through multiple iterations as new codes emerged and was used to index the remaining transcripts. A matrix was generated using a spreadsheet, where the data were charted. Charting involved referencing interesting or illustrative quotations from each transcript under the identified codes. During the mapping and interpretation stage, the matrices were closely examined to make comparisons within categories across participants and between participants across categories. Memos were written for each category and discussed with the team. We used an iterative process of data collection and analysis.

Throughout the data collection and analysis process, team members engaged in reflexive dialogue, with coding conducted by one researcher and interpretations discussed iteratively within the research team to refine themes. The framework method facilitated a clear audit trail from the original raw data in transcripts to the final themes, including the illustrative quotes. To maintain clarity and avoid overlength in the main text, additional illustrative participant quotations are provided in Additional File 3.

*Vaccine acceptance* refers to a positive attitude toward vaccination and expressed willingness to receive the vaccine. *Vaccine uptake* refers to the actual receipt of the vaccine. *Vaccine hesitancy* refers to uncertainty or delays in deciding to vaccinate despite vaccine availability, often reflecting concerns about safety, necessity, or trust. *Vaccine refusal* refers to a deliberate decision not to receive the vaccine when it is available and accessible.

## Results

### Study population

A total of 89 individuals participated in fifteen FGDs, with ages ranging from 12 to 75 years (median: 28, IQR: 18 to 36). The characteristics of the participants are detailed in Table 1. We were unable to conduct FGDs with vaccinated adolescent girls due to difficulties in mobilising them for the discussions.

**Table 1:** Demographic characteristics of the participants in the focus group discussions (*n* = 89)

Characteristic	No	%
**Age**		
12–20	29	32.6%
21–30	24	27.0%
31–40	23	25.8%
> 41	13	14.6%
**Gender**		
Male	33	37.1%
Female	56	62.9%
**Education**		
Professional degree	2	2.2%
Graduate or postgraduate	9	10.1%
Intermediate or post high school diploma	22	24.7%
High school certificate	21	23.6%
Middle school certificate	12	13.5%
Primary school certificate	17	19.1%
No formal education	6	6.7%
**Occupation**		
Semi-professional	2	2.2%
Clerks/shops/farms	2	2.2%
Skilled work	8	9.0%
Semi-skilled work	1	1.1%
Unskilled	11	12.4%
Not gainfully employed	1	1.1%
Housewife	37	41.6%
Student	27	30.3%
**Religion**		
Hindu	63	70.8%
Muslim	23	25.8%
Christian	3	3.4%
**Monthly family income**		
< 5000	18	20.2%
5001–10000	28	31.5%
10,001–15000	20	22.5%
15,001–20000	12	13.5%
> 20,000	9	10.1%
no data	2	2.2%

The FGDs averaged 40 min with approximately six participants per session, and details are shown in Table 2.

**Table 2:** Characteristics of the focus group discussions (*n* = 15)

FGD No	TCV vaccination status	FGD Category	Gender	No. of participants
FGD 1^*^	Unvaccinated	Guardian (1–11)	Female	6
FGD 2^*^	Vaccinated	12 to 17	Male	7
FGD 3	Unvaccinated	Guardian (12–17)	Female	5
FGD 4	Unvaccinated	12 to 17	Male	6
FGD 5	Vaccinated	18 to 30	Male	6
FGD 6	Unvaccinated	18 to 30	Male	5
FGD 7	Vaccinated	Guardian (12–17)	Female	6
FGD 8	Unvaccinated	18 to 30	Female	6
FGD 9	Unvaccinated	Guardian (1–11)	Female	5
FGD 10	Vaccinated	Guardian (1–11)	Female	8
FGD 11	Vaccinated	18 to 30	Female	6
FGD 12	Unvaccinated	12 to 17	Female	7
FGD 13	Vaccinated	Head of the household	Male	6
FGD 14	Vaccinated	18 to 30	Male	3
FGD 15	Vaccinated	Guardian (1–11)	Female	7

^*^Pilot FGDs

Additionally, five IDIs were conducted, the details of which are listed in Table 3.

**Table 3:** Demographic characteristics of the participants in the in-depth interviews (*n* = 5)

IDI No	Age	Gender	Category	Education	Occupation	Monthly family income	Religion
IDI 1	41	Male	Head of the household	Graduate	Semi-skilled	12,000	Christian
IDI 2	60	Male	Head of the household	High school	Retired	20,000	Muslim
IDI 3	32	Male	Head of the household	Graduate	Clerks/shops/farms	12,000	Muslim
IDI 4	13	Female	12 to 17	Primary	Student	7000	Muslim
IDI 5	14	Female	12 to 17	Primary	Student	12,000	Muslim

All participants included in the in-depth interviews (IDIs) were unvaccinated

### Themes

We identified six themes from thematic analysis: (1) Impact of COVID-19 (2) Fear of immediate adverse events (3) Perceived risk of disease (4) Influence of the social circle (5) Role of trust and confidence in the healthcare system (6) Role of benefits from the study.

### Impact of COVID-19

#### *Fear of long-term harms*

Negative experiences with COVID-19 vaccination impacted TCV uptake, as participants feared similar long-term effects, such as body aches, joint pain, and fatigue, which they attributed to the COVID-19 vaccine. They shared both their own experiences and those they heard from others and in news reports, which heightened their anxiety and led to a broader fear of vaccines.

*"We became very dull after taking the COVID vaccine. On top of that, the thought of giving this [vaccine] to our children makes us feel bad. We are struggling ourselves, and we don't want our children to face the same challenges tomorrow. Since getting vaccinated, our limbs have been hurting, and we feel weak." *

*[Mother of unvaccinated child,37 years, FGD 9]*

Some unvaccinated adult participants expressed concerns that potential side effects might not be known immediately, citing examples like those later announced by COVID-19 vaccine manufacturers. They also stated that they require proof of the vaccine's quality before considering vaccination, particularly regarding its manufacturing process and approval. Many mothers of unvaccinated children mentioned that there was no "guarantee" given to assure them that nothing adverse would happen.

#### *Shift in attitudes toward new and adult vaccination*

Many unvaccinated participants expressed scepticism about new vaccines, preferring to stick with routine vaccinations. Additionally, they were doubtful about the expanding age range for vaccination, stating that vaccines have traditionally been administered until age 15, and they are uncertain about the rationale behind the recent introduction of vaccines for adults. They believe that this shift began following the introduction of the COVID-19 vaccine.

*"Similarly, they are administering many vaccines at nine months, five months, and are advising vaccinations at three months. Vaccines are being given regularly. Now, if we talk about vaccines for adults... then why is that?" *

*[Guardian of unvaccinated children, Female,50 years, FGD 3]*

#### *Free vaccines and perceived quality*

The typhoid vaccine is usually available only in private hospitals for a fee and is not yet included in the government immunisation schedule in India. During the campaign, the vaccine was explicitly communicated as being free of charge. However, many participants expressed doubts about the quality of the vaccine offered for free, particularly after receiving the COVID-19 vaccine at no cost. They questioned why the typhoid vaccine was free when it usually requires payment elsewhere.

Conversely, mothers from more socioeconomically disadvantaged areas appreciated free access, stating that they might not have been able to obtain the vaccine otherwise. Many mothers suggested that if the vaccine was offered at a nominal price and that they were convinced of its efficacy, they would be more likely to accept it.

*"If we need to see a doctor, we have to pay a fee of 300 rupees. So, if the vaccine costs 200 rupees, we can go and get it. If it’s under 500 rupees, we would be willing to pay since it’s beneficial for the children."*

*[Mother of vaccinated child,40 years, FGD 15]*

#### *Increased disease salience post-pandemic*

Many mothers of vaccinated children shared that their experience with COVID-19 made them realise that unexpected diseases could arise, and they were concerned about avoiding a similar situation. This motivated them to ensure their children were vaccinated.

*"We wouldn't have taken it [the vaccine]. Because of the occurrence of a disease like COVID and the awareness that another disease shouldn't impact us in the same way, people came forward to get vaccinated."*

*[Mother of vaccinated adolescent,35 years, FGD 7]*

### Fear of immediate adverse events

Fear of immediate adverse events like pain and fever is a common reaction to vaccination, often based on past experiences. Adolescents, who often perceive themselves as adults, more openly expressed concerns about pain and the number of injections compared with younger children. A key barrier for them is fear of needles, combined with incomplete information from news and social circles, leading to vaccine refusal. Having received the Td (Tetanus and Diphtheria) vaccine at 15 years and two doses of the COVID-19 vaccine, they felt the typhoid vaccine was excessive.

*"That is, every year they keep saying we need to take vaccines, and also they mention it in school. They told us in tenth grade that we should get it, so we did. Then it came up again in eleventh and twelfth grade. If we keep getting injections year after year, what will happen to our bodies? So, we will say no and let it go."*

*[Unvaccinated adolescent,17 years, Male, FGD 4]*

Similarly, parents and adolescents often postpone or avoid vaccination due to upcoming examinations. They fear that potential sick days or pain from the vaccination could disrupt their study schedules and exam preparation.

*“Our daughter was studying in 10th grade, so we skipped it because the exams were happening.”*

*[Mother of unvaccinated adolescent,40 years, FGD 1]*

Adult women expressed concerns that vaccination might disrupt their daily responsibilities, fearing pain and fever could hinder their ability to manage household tasks and care for their families. Some mothers chose to vaccinate their children first and monitor their reactions before getting vaccinated themselves. Adult men expressed concerns that the headaches and fever they experienced after the COVID-19 vaccine made them feel the discomfort was worse than the disease threat. This fear of pain has contributed to their hesitancy towards other vaccines.

Many people also believed that pre-existing conditions such as diabetes and cardiovascular diseases are contraindications for vaccination.

### Perceived risk of disease

Some individuals do not perceive typhoid as an immediate threat, primarily due to a lack of awareness about its prevalence in their community. This perceived low risk leads them to believe that vaccination is unnecessary, as they do not observe the disease affecting those around them.

*“Now you’re talking about typhoid. Even if you ask how many people in this street had it[typhoid], we don’t know. If I don’t know about it, why should I take the injection for a disease that isn’t present?”*

*[Unvaccinated adult, Male,27 years, FGD 6]*

Most adult males perceived themselves as healthy and not susceptible to typhoid, believing that vaccination is intended for children. Additionally, some participants expressed misperceptions that typhoid is a self-limiting illness and is not severe enough to justify vaccination.

Many adolescents shared similar perceptions, stating they were uninformed about the importance of vaccination. They believe they should be more involved in vaccination discussions, as neither the study team nor their schools provide details about the vaccines offered or their purposes, leaving only parents informed.

On the other hand, many mothers of vaccinated children viewed the typhoid vaccine as essential, and they were conscious that poor sanitation increases the likelihood of infection. These individuals recognised the vulnerability created by unhygienic conditions and were motivated to protect themselves and their families.

*"I decided to take it mainly because there is a lack of hygiene here. Given that, I thought that taking this vaccine would definitely be beneficial. That was my mindset: if we take this, we won't get infected by that disease."*

*[Vaccinated adult, Female,30 years, FGD 11]*

Many mistakenly believed typhoid is caused by mosquitoes and suggest preventing it by avoiding stagnant water in tires, tanks, fridge trays, and containers. Some vaccinated individuals felt at risk due to the high presence of mosquitoes, and past experiences with dengue motivated them to get vaccinated. They requested the introduction of dengue and chikungunya vaccines, citing concerns about open drains and mosquitoes, and felt the typhoid vaccine should have been introduced earlier.

### Influence of the social circle

#### *Objection from decision makers of the family*

In many households, women mentioned that they rely on their husbands for decisions regarding vaccinations, and if their husbands disagree, they would refrain from getting their children vaccinated, fearing repercussions if any adverse events occur.

*"We ask our husbands first. If they say no, we don’t take it. If they say yes, then we go ahead and take it."*

*[Mother of unvaccinated child,29 years, FGD 8]*

#### *Observing and listening to others’ vaccination choices*

Some individuals reported negative influences from friends or relatives who claimed that the vaccine could cause typhoid. Additionally, a common misconception among some segments of the Muslim community was that vaccines could cause infertility. Some individuals have raised concerns that vaccines might disrupt puberty or fertility, fearing that this could threaten their community. Some mothers advised their adolescent children to decline school-offered vaccines once they reached puberty.

*"They say that if the injection is taken, the baby won't be born. It will cause problems, and if we give it to young girls, they won’t reach puberty. That’s what people are saying… that injections shouldn’t be taken. We hear this from others, you know, when they talk at the shops, saying things like, ‘They gave this injection, but it shouldn’t be taken’.”*

*[Mother of unvaccinated child,45years, FGD 3]*

Several mothers of unvaccinated children reported that their husbands or friends believe vaccines should only be administered when a child has a fever, reflecting a prevalent misconception about vaccination necessity. Overall, misconceptions related to fertility and that vaccines are only required when children have a fever were more commonly mentioned among mothers of unvaccinated participants in low coverage areas.

On the other hand, neighbours who recognised the benefits of vaccination encouraged those around them by showcasing their children's good health after vaccination and reinforcing the belief that vaccines prevent diseases. Moreover, many vaccinated participants reported initial hesitancy, opting to wait and observe others' responses to the vaccine. Seeing others get vaccinated without adverse effects boosted their confidence, ultimately leading them to get vaccinated as well.

*"At first, I didn’t believe what that brother (field worker) said, so I didn’t take it either. It was only after I saw a few others get vaccinated without any issues that I decided to take my children and get them vaccinated too."*

*[Mother of vaccinated child,35 years, FGD 10]*

Most adolescents accepted vaccination based on their mothers' instructions or their friends' influence.

### Role of trust and confidence in the healthcare system

#### *Trust in the vaccine provider*

Although PHCs were used as vaccination centres, some individuals expressed distrust in the vaccination delivery process due to the perceived lack of visible government involvement. They believed that if the vaccine was truly important, the government would include it in the immunisation schedule, leading to doubts about its necessity. The fact that the vaccine was provided by a private institution, increased their scepticism. The absence of government health workers further increased their doubts.

*"We'll only take it if the government conducts it because they'll follow the proper rules. If it’s private, we don’t know who’s involved or what’s being done."*

*[Mother of unvaccinated child,36 years, FGD 9]*

Conversely, a few mothers preferred private treatment, feeling that government medicines were substandard. Many participants had long-standing trust in the hospital operated by the community health department of the study institution, where they received antenatal care, delivery services, and vaccinations for their children. This trust motivated many to get vaccinated, as they believed the vaccine was both high-quality and essential.

Some unvaccinated individuals expressed disinterest in vaccines, viewing them as outside traditional medicine and placed their trust in alternative treatments, particularly Ayurveda. They questioned the safety and production processes of vaccines, citing a lack of knowledge about how they are made. Additionally, some participants mentioned avoiding all vaccines in the immunisation schedule due to distrust in modern medicine.

#### *Trust in healthcare professional recommendations*

Many participants who chose to get vaccinated emphasised the importance of trust in healthcare professionals. They were particularly motivated by doctors and nurses who visited their homes to educate them on the benefits of vaccination.

*“At home, the doctor came and said we could take it, and that’s when we decided to go ahead. We didn't ask anyone else."*

*[Mother of vaccinated adolescent,49 years, FGD 7]*

Many of the participants mentioned that they consulted with medical professionals, such as their family doctor or a nurse relative, before deciding to receive the vaccine. This consultation provided them with the assurance they needed, as they trusted the expertise of these professionals and believed that the vaccine would effectively prevent disease.

#### *Feelings of safety and assurance*

The study includes a monthly follow-up component where a fieldworker visits each participant's home to verify their residence, monitor any migration and fever illnesses in the previous month. They build rapport with the community and inquire about the wellness of the participants. Many participants reported that this consistent interaction contributed to a feeling of safety as they had a direct point of contact in case of any adverse events, which in turn motivated them to get vaccinated.

*“They come every month and ask about the children. They inquire if there has been any fever, cold, or anything else in the past month. They check on us at least once or twice a month, and it feels good to have someone looking out for the children. Their regular visits make us feel safe. That is why”*

*[Mother of vaccinated children,36 years, FGD 7]*

### Role of benefits from the study

Travel assistance to vaccination sites, along with house-to-house efforts, boosted participation. The study reimburses healthcare and travel costs for all fever-related illnesses. Participants received a card with their name, unique ID, and a helpline number for accessing health facilities. Those who called the helpline before visiting were reimbursed for their healthcare expenses, and a nurse or fieldworker from the study followed up with them until they were fever-free for three days. These components motivated participants to get vaccinated, as they appreciated the easy access to free healthcare and valued the attention to their health.

However, a few participants expressed scepticism about the benefits offered, noting that it was uncommon for anyone to come to their homes to administer vaccines, which led them to question the intentions behind such generous offers.

**Fig. 2:**
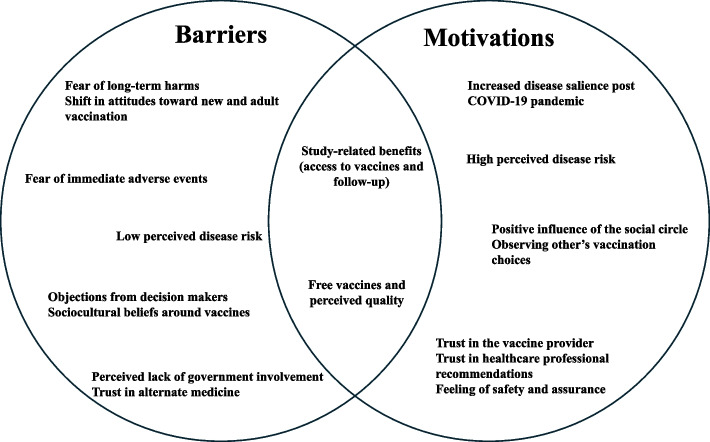
Conceptual representation of barriers and motivations influencing typhoid conjugate vaccine (TCV) uptake. Factors shown on the left functioned primarily as barriers, those on the right as motivations, and factors in the overlapping area operated bidirectionally depending on context, particularly study-related benefits such as access to vaccination and follow-up

Figure 2 synthesises how the identified sub-themes interacted to shape decisions around TCV uptake, clustering as barriers, motivations, or bidirectional influences rather than operating in isolation. Barriers were primarily related to safety concerns, low perceived disease risk, and social or institutional mistrust, while motivations were driven by heightened disease salience following the COVID-19 pandemic, perceived susceptibility, positive social influence, and trust in healthcare providers. Study-related benefits, including free vaccination and improved access, operated bidirectionally, facilitating uptake for many while generating suspicion among a few, highlighting the context-dependent nature of vaccine decision-making.

## Discussion

This qualitative study explored factors influencing typhoid vaccine uptake among participants in a clinical trial in Vellore, India. The findings highlight a complex interplay of individual beliefs, household dynamics, and socio-cultural factors that act as both barriers and motivators. Barriers included misconceptions about vaccine safety, fear of needles, perceived low risk of typhoid, gender dynamics within households, and cultural beliefs. Motivators included trust in healthcare providers, desire for self and family protection, social influence, long-term monitoring, and the programme’s convenience and affordability. While many participants expressed concerns about the long-term safety of new vaccines due to their experiences with COVID-19 vaccination [10, 22] the unpredictability of the virus motivated others, especially mothers, to vaccinate their children as a precaution against future diseases.

Fear of needles, pain, and post-vaccination sick days were major barriers, especially among adolescents and adults, which is consistent with other studies [23]. Many adults were concerned about disruptions to their daily activities. This highlights how both physical discomfort and the fear of lost productivity shape vaccine hesitancy in adults. A key finding was the difference in risk perception between vaccinated and unvaccinated participants. Vaccinated individuals felt susceptible to typhoid, while the unvaccinated individuals believed they were not at risk. This aligns with previous studies [16, 24], which identifies perceived susceptibility and perceived risk as key drivers of vaccination. Our findings reveal a significant knowledge gap about typhoid, its transmission, and risks, particularly in endemic areas like Vellore.

Social circles, including community and household dynamics, significantly influenced vaccine uptake both positively and negatively. On the positive side, many vaccinated participants were motivated by friends, neighbours, and the broader community, echoing findings from Vietnam [25]. Conversely, gender dynamics within households adds another layer of complexity to vaccine decision-making. The husband or male elder, who often serves as the primary decision maker, frequently missed team visits where key vaccine information was shared because of work-related constraints, typically leaving home early and returning late at night. Although outreach activities were conducted on Sundays, and this was communicated in advance, reaching male decision-makers consistently remained challenging. Women, while acknowledging the importance of vaccination, faced challenges in exercising agency in family healthcare decisions, reducing the likelihood of children being vaccinated. From a trial implementation perspective, our findings emphasise the need for vaccination campaigns that engage both men and women [26, 27]. Additionally, misconceptions about vaccine safety, particularly fears of infertility in the Muslim community, emerged as a significant barrier to typhoid vaccine uptake, a fear that has persisted across various settings [13]. Such fears are not unique to typhoid vaccination and have historical precedence, including rumours during vaccination campaigns in Cameroon in the 1990 s that vaccines were intended to cause infertility or reduce fertility among targeted populations [28]. Research links belief in vaccine conspiracy theories to lower acceptance rates, often tied to higher religiosity [29]. Hesitancy was evident in a Muslim-majority area where FGDs couldn't be conducted due to participant reluctance. To mitigate this, we conducted IDIs in these areas and held FGDs in locations with similar sociodemographic characteristics; however, the most hesitant groups may still have been missed. Our findings align with other studies [30, 31] highlighting the need for culturally sensitive education from trusted sources and engaging religious leaders.

Interestingly, the provision of free vaccines yielded mixed responses. While many appreciated the increased accessibility, some perceived free vaccines as being of lower quality, suggesting that a nominal fee might increase their motivation to get vaccinated. A willingness-to-pay study from Vietnam advocates a subsidised price for typhoid vaccines in mass campaigns to allow partial cost recovery, even with limited public funding [32]. Furthermore, a Kuwait study revealed offering HPV vaccines for free decreased vaccination likelihood [33], indicating that entirely free interventions may be perceived as lower quality, especially where out-of-pocket expenses are common. Exploring alternative models, like subsidised pricing or linking vaccination to valued services, may help mitigate this perception and boost uptake.

Despite existing barriers, several strong motivators for vaccine uptake emerged, particularly trust in healthcare providers. Previous research indicates that healthcare professionals are trusted sources of health information [34], and their recommendations significantly influence health behaviours. Investing in training healthcare workers to communicate effectively about vaccines and address concerns can greatly enhance vaccine uptake.

Long-term monitoring is vital in building trust and encouraging vaccination. Regular check-ups, accessible reporting systems for adverse events, and clear communication can enhance trust and address safety concerns. Furthermore, the convenience and affordability of the vaccination programme with convenient clinic locations, flexible hours, transportation facilities, and minimal out-of-pocket expenses along with the desire to protect themselves and their families from typhoid, motivated many to get vaccinated.

Our findings highlight the need to establish realistic goals and allocate sufficient time to build awareness and trust, rather than rushing mass vaccination campaigns in an attempt to achieve high coverage. When viewed through established behavioural frameworks, the identified themes align with core constructs relevant to vaccine decision-making. Perceived risk of disease, fear of immediate and future adverse events, and attitude toward new and adult vaccines correspond to perceived susceptibility and severity within the HBM [16] and to the “thinking and feeling” domain of the WHO BeSD framework [17]. Social influences, including household decision-making and observing others’ vaccination choices, reflect key social processes shaping motivation. Trust in healthcare professional recommendations aligns with confidence in vaccines and health systems, while study-related benefits and views on free vaccine availability relate to practical access and service-related considerations. This framing helps distinguish barriers, motivators, and contextual conditions, and highlights intervention opportunities beyond logistics alone. They emphasise the need to address both individual ('thinking and feeling') and social ('social processes') factors influencing vaccination decisions, and not just practical barriers.

Our study addresses the critical gap in understanding the factors influencing vaccine uptake beyond routine immunisation in India. Identifying these drivers is essential for overcoming challenges in mass immunisation campaigns and introducing new vaccines. The qualitative nature of our study limits the generalisability of our findings. The total number of participants in the upper income quintile was small, and thus, caution should be taken in interpreting the findings or generalising to larger subgroups. While linkage to the trial is a key strength, trial-specific features such as intensive follow-up, participant support through helpline, reimbursement, and institutional credibility may have enhanced trust and perceived safety, potentially limiting generalisability to routine programme settings. At the same time, trial settings may heighten attention to safety due to the experimental framing of interventions; however, in this study, such concerns were likely mitigated by the fact that TCV is a licenced vaccine with a proven safety profile and is currently available in the market, with endorsement from national bodies such as the Indian Academy of Paediatrics (IAP). While specific findings may not be generalizable beyond the study context, the thematic areas of importance to typhoid vaccine may resonate elsewhere, and they can guide thinking about creating robust vaccination programmes in other jurisdictions. Further research with a larger and more diverse sample size is needed to confirm and expand upon these findings.

## Conclusion

This study highlights how typhoid vaccine uptake is shaped by a complex interplay of perceptions, social dynamics and trust. In the post-pandemic era, vaccine availability alone does not ensure uptake; even when vaccines are accessible and free, uptake depends on community engagement and trust. Our findings identify several considerations relevant to the design and conduct of vaccine trials and vaccination programmes, including heightened expectations for safety assurance following the COVID-19 pandemic, adult-specific concerns related to pain, productivity loss and perceived necessity of vaccination, the influence of household decision-making dynamics, and the importance of visible government involvement and trusted institutions. Together, these findings suggest that successful trial implementation and vaccine rollout require not only effective delivery systems, but also transparent communication, engagement of healthcare providers and key decision makers, and deliberate efforts to build community readiness alongside logistical planning.

## Supplementary Information

Additional file 1: Consolidated criteria for reporting qualitative studies (COREQ): 32-item checklist.

Additional file 2: Thematic guide for the interviews.

Additional file 3: Additional participant quotations under identified themes and subthemes for barriers and motivations to vaccine uptake.

## Data Availability

The datasets used and/or analysed during the current study are available from the corresponding author on reasonable request.

## Abbreviations

BeSD: Behavioural and Social Drivers
CMC: Christian Medical College
FGD: Focus Group Discussions
HBM: Health Belief Model
IDI: In-depth Interviews
PHC: Primary Health Centres
TCV: Typhoid Conjugate Vaccine
WASH: Water, Sanitation and Hygiene
WHO: World Health Organisation
