# Supplementary material for: Exploring barriers to and motivations for vaccine uptake in a typhoid vaccine trial in Vellore, South India: a qualitative study
Source: BMC Public Health. 2026 Jan 31;26:760. doi: 10.1186/s12889-026-26362-z (PMC12952106; doi:10.1186/s12889-026-26362-z)
Supplement: Supplementary file 2 — Additional file 2: Thematic guide for the interviews. [file 12889_2026_26362_MOESM2_ESM.pdf]

# Thematic guide for Focus group discussions/In-depth interviews

## Vaccinated participants

1. **Can you share your understanding about vaccines in general?**
   Probes:
   a) In your perspective, what do you feel is the right age for administering vaccines, and why?
   b) Who in your family makes the decision about receiving vaccinations?

**2. What is your understanding about typhoid fever?**

Probes: how is it contracted, its symptoms?

a) could you share personal experiences if you or your family members have been affected by typhoid fever and what preventive measures can be taken to prevent the risk of typhoid fever?

**3. Have you heard about typhoid vaccine? What have you heard? Where?**

**4. What are the various reasons for deciding to get vaccinated?**

Probes: Where or to whom would you seek information before deciding to get vaccinated?

a) Can you share any discussions in your family or social circle regarding vaccination?
b) Would you recommend this vaccine to others? If so, what reasons would you give for your recommendation?

**5. Were there any challenges or barriers that you've faced in accessing the vaccine?**

Probes:

a) Could you share the accessibility of vaccination sites, the availability of transportation to these sites, and the information regarding their locations and operating hours?
b) Have you received any support or assistance from the team to access the vaccine?

**6. Are there any cultural beliefs or social norms related to vaccine that influence your decision to get vaccinated?**

a) Have you faced any stigma or discrimination related to receiving vaccine?

7. What are your current thoughts on the decision you've made regarding vaccination? Why?

8. Is there anything else you would like to share about your experiences or perspectives about the vaccine?

## Non-vaccinated participants

1. **Can you share your understanding about vaccines in general?**
   Probes:
   a) In your perspective, what do you feel is the right age for administering vaccines, and why?
   b) Who in your family makes the decision about receiving vaccinations?

**2. What is your understanding about typhoid fever?**

Probes: how is it contracted, its symptoms?

a) could you share personal experiences if you or your family members have been affected by typhoid fever and what preventive measures can be taken to prevent the risk of typhoid fever?

**3. Have you heard about typhoid vaccine? What have you heard? Where?**

**4. Could you please tell us what are the reasons that prevented you from getting vaccinated?**

Probes: Where or to whom would you seek information regarding vaccination?

a) Can you share any discussions in your family or social circle regarding vaccination? Any personal reasons?
b) Can you share any past experiences regarding vaccines that influenced your decision to get vaccinated?

**5. Were there any logistical challenges or barriers that prevented you from accessing the typhoid vaccine?**

Probes: vaccination site accessibility, transportation to site, information regarding vaccination site locations and operating hours?

a) Have you received any support or assistance from the team to access the vaccine?

**6. Are there any cultural beliefs or social norms related to vaccine that influence your decision?**

a) Were there any concerns or fears about potential stigma or discrimination for not getting vaccination?

7. What are your current thoughts on the decision you've made regarding vaccination? Why?

8. Is there anything else you would like to share about your experiences or perspectives on the vaccine?
