# Supplementary material for: Exploring barriers to and motivations for vaccine uptake in a typhoid vaccine trial in Vellore, South India: a qualitative study
Source: BMC Public Health. 2026 Jan 31;26:760. doi: 10.1186/s12889-026-26362-z (PMC12952106; doi:10.1186/s12889-026-26362-z)
Supplement: Supplementary file 3 — Additional file 3: Additional participant quotations under identified themes and subthemes for barriers and motivations to vaccine uptake. [file 12889_2026_26362_MOESM3_ESM.docx]

**Themes and subthemes for barriers and motivations to vaccine uptake in a typhoid vaccine clinical trial**

| **Themes** | **Subthemes** | **Sample quotes** |
| --- | --- | --- |
| Impact of COVID-19 | Fear of long-term harms | "We became very dull after taking the COVID vaccine. On top of that, the thought of giving this [vaccine] to our children makes us feel bad. We are struggling ourselves, and we don't want our children to face the same challenges tomorrow. Since getting vaccinated, our limbs have been hurting, and we feel weak." [Mother of unvaccinated child, 37 years, FGD 9]  "There's a lot of talk about vaccinated people having heart attacks, and I’m not sure if those rumours are true. It creates a fear that something similar could happen to us. We haven't vaccinated our children yet, and now if I decide to go ahead with it, we won't know the long-term effects until maybe ten years down the line. What if they say, 'No, sir, this is just a research study'? You might argue that anything can happen in research. They could claim that only a small number of kids—maybe two out of a hundred—might be affected. But what if those two kids were mine? This is just my personal opinion." [IDI 3] |
|  | Shift in attitude towards adult and new vaccination | "Similarly, they are administering many vaccines at nine months, five months, and are advising vaccinations at three months. Vaccines are being given regularly. Now, if we talk about vaccines for adults... then why is that?" [Guardian of unvaccinated children, Female, 50 years, FGD 3]  “Now, even after the corona vaccine, the fever came. What guarantee is there that this [typhoid] won’t come after taking this [vaccine]?"[Adolescent girl, FGD 12] |
|  | Free vaccines and perceived quality | "Anything offered for free can be a bit concerning. If they set a specific price, we would think of it as a good quality. After experiencing issues with the COVID vaccine, we felt hesitant about taking another vaccine if it was free. So, we decided to wait for a while before getting it." [Vaccinated participant, Female, 28 years, FGD 11]  "If we need to see a doctor, we have to pay a fee of 300 rupees. So, if the vaccine costs 200 rupees, we can go and get it. If it’s under 500 rupees, we would be willing to pay since it’s beneficial for the children." [Mother of vaccinated child,40 years, FGD 15] |
|  | Pandemic as a motivator | "We wouldn't have taken it [the vaccine]. Because of the occurrence of a disease like COVID and the awareness that another disease shouldn't impact us in the same way, people came forward to get vaccinated." [Mother of vaccinated adolescent, 35 years, FGD 7] |
| Fear of immediate adverse events |  | "That is, every year they keep saying we need to take vaccines, and also they mention it in school. They told us in tenth grade that we should get it, so we did. Then it came up again in eleventh and twelfth grade. If we keep getting injections year after year, what will happen to our bodies? So, we will say no and let it go." [Unvaccinated adolescent, Male, 17 years, FGD 4]  “Our daughter was studying in 10th grade, so we skipped it because the exams were happening.” [Mother of unvaccinated child, 40 years, FGD 1]  "After the children get vaccinated, if they don't experience any side effects for a few days and are fine, then we will go and get vaccinated ourselves. We have household work to do, right? We are the housewives. So, we shouldn't get any fever, isn’t it? That's how we do it in our home. Yeah! It usually happens that way." [Vaccinated participant, Female, 26 years, FGD 11]  "I took the first dose of Covishield, and the fever that came at that time made me feel like getting COVID would’ve been better...[I] didn’t take the second dose, not because of fear, but because of the impact it [first dose] had on me." [Unvaccinated adult, Male, FGD 6] |
| Perceived risk of disease |  | “Now you’re talking about typhoid. Even if you ask how many people in this street had it[typhoid], we don’t know. If I don’t know about it, why should I take the injection for a disease that isn’t present?” [Unvaccinated adult, male, 27 years, FGD 6]  "When it comes to injections at a young age, like ten or fifteen, they are given to everyone under their mother's control. However, once they start to think for themselves, it's common for them to say no. As they reach a certain stage and begin to think independently, they feel that it's not necessary." [Unvaccinated adult, male, FGD 6]  "I decided to take it mainly because there is a lack of hygiene here. Given that, I thought that taking this vaccine would definitely be beneficial. That was my mindset: if we take this, we won't get infected by that disease." [Vaccinated adult, Female, 30 years, FGD 11]  "I had dengue fever when I was pregnant with my daughter, ma'am.. Because I had it, I want to make sure the children don’t get it. Whatever vaccine is available for children, I always make sure to take it." [Mother of vaccinated child, 38 years, FGD 15] |
| Influence of the Social Circle | Objection from decisionmakers of the family | "We ask our husbands first. If they say no, we don’t take it. If they say yes, then we go ahead and take it." [Unvaccinated adult, Female, 29 years, FGD 8] |
|  | Observing and listening others’ vaccination choices | "They say that if the injection is taken, the baby won't be born. It will cause problems, and if we give it to young girls, they won’t reach puberty. That’s what people are saying… that injections shouldn’t be taken. We hear this from others, you know, when they talk at the shops, saying things like, ‘They gave this injection, but it shouldn’t be taken’.” [Mother of unvaccinated adolescent, 45 years, FGD 3]  "Someone nearby took the vaccine and said, 'Nothing happened to my child; he’s perfectly fine.' Hearing that, we felt reassured. Initially, we were scared and decided not to take it—we didn’t want it. I avoided it for a month. But after they told us, 'Nothing happened, the children are fine, you should go and take it too. Don’t be scared,' we felt confident." [Mother of vaccinated adolescent,39 years, FGD 7] "At first, I didn’t believe what that brother said, so I didn’t take it either. It was only after I saw a few others get vaccinated without any issues that I decided to take my children and get them vaccinated too." [Mother of vaccinated child, 35 years, FGD 10] |
| Role of trust and confidence in the healthcare system | Trust in the vaccine provider | "We'll only take it if the government conducts it because they'll follow the proper rules. If it’s private, we don’t know who’s involved or what’s being done." [Mother of unvaccinated child, 36 years, FGD 9]  “Before this, no one ever came and specifically advised anyone to take something like this. It was the doctors who took the initiative, recognizing that many people are getting typhoid and that we should prevent it. They arranged for this for the community. [institution name] is well-known in this area, and there’s a belief that if we go there, we will receive proper treatment.” [Vaccinated adult, Female, 28 years, FGD 11]  "Trust is more on Ayurveda. But with English medicine, people don't have that same confidence. We don't know how it's made, what tests are done, or what the results are. Since we don’t know, we worry that something might happen to us if we take it. If anything has happened to others, we automatically think it could happen to us too. So, when it's offered, we decide not to take it." [Unvaccinated adult, Male, 22 years, FGD 6] |
|  | Trust in healthcare professional recommendations | “At home, the doctor came and said we could take it, and that’s when we decided to go ahead. We didn't ask anyone else." [Mother of vaccinated adolescent, 49 years, FGD 7]  “My sister-in-law is a nurse. We asked her. Here there's a sister who is a nurse. We asked her as well. They said you can take, it's not a problem.” [Mother of vaccinated child, 49 years, FGD 10] |
|  | Feeling of safety and assurance | “They come every month and ask about the children. They inquire if there has been any fever, cold, or anything else in the past month..They check on us at least once or twice a month, and it feels good to have someone looking out for the children. Their regular visits make us feel safe. That is why.” [Mother of vaccinated adolescent, 36 years, FGD 7] |
| Role of benefits from the study |  | “Using the card is just the best. It has reduced our medical expenses. Last time we took our older daughter, they didn't charge us much—it was free. They also ask us to call before we go, and even if we call at midnight, they pick up the phone. They check in with us, asking how our health is and how the children are doing. All of this is really great.” [Head of the household of vaccinated participants, Male, 33 years, FGD 13] |
